# A Novel Rat Model of Cardiac Donation After Circulatory Death Combined With Normothermic *ex situ* Heart Perfusion

**DOI:** 10.3389/fcvm.2021.639701

**Published:** 2021-07-23

**Authors:** Jiale Li, Chuqing Xue, Xiao Ling, Yu Xie, Desai Pavan, Huimin Chen, Qinbao Peng, Shaoyan Lin, Kunsheng Li, Shaoyi Zheng, Pengyu Zhou

**Affiliations:** ^1^Department of Cardiovascular Surgery, Nanfang Hospital, Southern Medical University, Guangzhou, China; ^2^Department of Cardiothoracic Surgery, Nanjing Drum Tower Hospital, Nanjing University Medical School, Nanjing, China

**Keywords:** normothermic *ex vivo* heart perfusion, donation after circulatory death, rat model, heart preservation, heart transplantation

## Abstract

**Background:** In heart transplantation, the adoption of hearts from donation after circulatory death (DCD) is considered to be a promising approach to expanding the donor pool. Normothermic *ex situ* heart perfusion (ESHP) is emerging as a novel preservation strategy for DCD hearts. Therefore, pre-clinical animal models of ESHP are essential to address some key issues before efficient clinical translation. We aim to develop a novel, reproducible, and economical rat model of DCD protocol combined with normothermic ESHP.

**Methods:** Circulatory death of the anesthetized rats in the DCD group was declared when systolic blood pressure below 30 mmHg or asystole was observed after asphyxiation. Additional 15 min of standoff period was allowed to elapse. After perfusion of cold cardioplegia, the DCD hearts were excised and perfused with allogenic blood-based perfusate at constant flow for 90 min in the normothermic ESHP system. Functional assessment and blood gas analysis were performed every 30 min during ESHP. The alteration of DCD hearts submitted to different durations of ESHP (30, 60, and 90 min) in oxidative stress, apoptosis, tissue energy state, inflammatory response, histopathology, cell swelling, and myocardial infarction during ESHP was evaluated. Rats in the non-DCD group were treated similarly but not exposed to warm ischemia and preserved by the normothermic ESHP system for 90 min.

**Results:** The DCD hearts showed compromised function at the beginning of ESHP and recovered over time, while non-DCD hearts presented better cardiac function during ESHP. The alteration of DCD hearts in oxidative stress, apoptosis, tissue energy state, histopathological changes, cell swelling, and inflammatory response didn't differ among different durations of ESHP. At the end of 90-min ESHP, DCD, and non-DCD hearts presented similarly in apoptosis, oxidative stress, inflammatory response, myocardial infarction, and histopathological changes. Moreover, the DCD hearts had lower energy storage and more evident cell swelling compared to the non-DCD hearts.

**Conclusion:** We established a reproducible, clinically relevant, and economical rat model of DCD protocol combined with normothermic ESHP, where the DCD hearts can maintain a stable state during 90-min ESHP.

## Introduction

Heart transplantation (HTx) has been introduced as the definitive management for patients with end-stage heart failure, however, this ultimate strategy has been hindered by the shortage of suitable donor hearts (1). Therefore, the adoption of hearts from donation after circulatory death (DCD) has been considered as a potential approach to expanding the donor pool (2).

However, DCD hearts inevitably suffer from myocardial injury following an obligatory warm ischemia time (from when the systolic blood pressure is lower than 50 mmHg after the withdrawal of life-sustaining therapy to reperfusion or cardioplegia) (3, 4). Furthermore, few cardioprotective therapies are allowed to apply in donors before circulatory death due to the ethical and legal issue (1, 3, 5, 6), and it's difficult to perform a proper functional assessment of a DCD heart before HTx (7).

Recently, normothermic *ex situ* heart perfusion (ESHP) is emerging as a promising preservation strategy for DCD hearts. This novel technique can perfuse donated hearts with warm, oxygenated, and nutrient-enriched blood-based perfusate in a semi-physiologic state, thereby reducing ischemic time, alleviating myocardial ischemia/reperfusion injury (IRI), prolonging donor heart preservation time, allowing a more objective evaluation for pre-transplant donor heart function, as well as offering a unique platform to repair DCD hearts *via* directly delivering post-conditioning agents, mesenchymal stem cells (MSCs) and MSCs-derived secretome into machine perfusion circuit (8, 9).

Before its efficient clinical translation, pre-clinical animal models of ESHP are essential to address some key issues, including ideal perfusion pressure, priming solution, and appropriate hematocrit, as well as efficacy and optimal dose of novel post-conditioning agents added into perfusion circuit (9, 10). Niederberger et al. suggested that pigs or rats appeared to be preferable for the pre-clinical model of DCD hearts, concerning clinically relevant ischemic tolerance (3). Although evolving as an ideal model to perform basic and translational researches about ESHP (11), the porcine model of ESHP has the obvious disadvantages of being costly and labor-intensive. On the other hand, a rat model of ESHP has some advantages over a porcine model as rats are less expensive, easier to handle and various disease models of rats are less challenging to be established. However, previous rat models of normothermic ESHP were not described in detail or clinically relevant enough (12–18).

Therefore, we set out to develop a clinically relevant and reproducible model of DCD protocol combined with normothermic ESHP in rats which can be used to address the aforementioned technical and practical issues in ESHP.

## Materials and Methods

### Animals

Male Sprague-Dawley rats (Charles River Laboratories, Beijing, China) used in this study received care in compliance with the Guide for the Care and Use of Laboratory Animals (National Institutes of Health Publication No. 85-23, revised 1996). All animal experiments were reviewed and approved by the Ethical Committee of the Laboratory Animal Research Center of Southern Medical University Nanfang Hospital. The rats were housed in temperature-controlled (22 ± 2°C) rooms with a 12-h light-dark cycle, given food and sterilized water, and acclimatized for 1 week.

### Experiment Design

**Figure 1:**
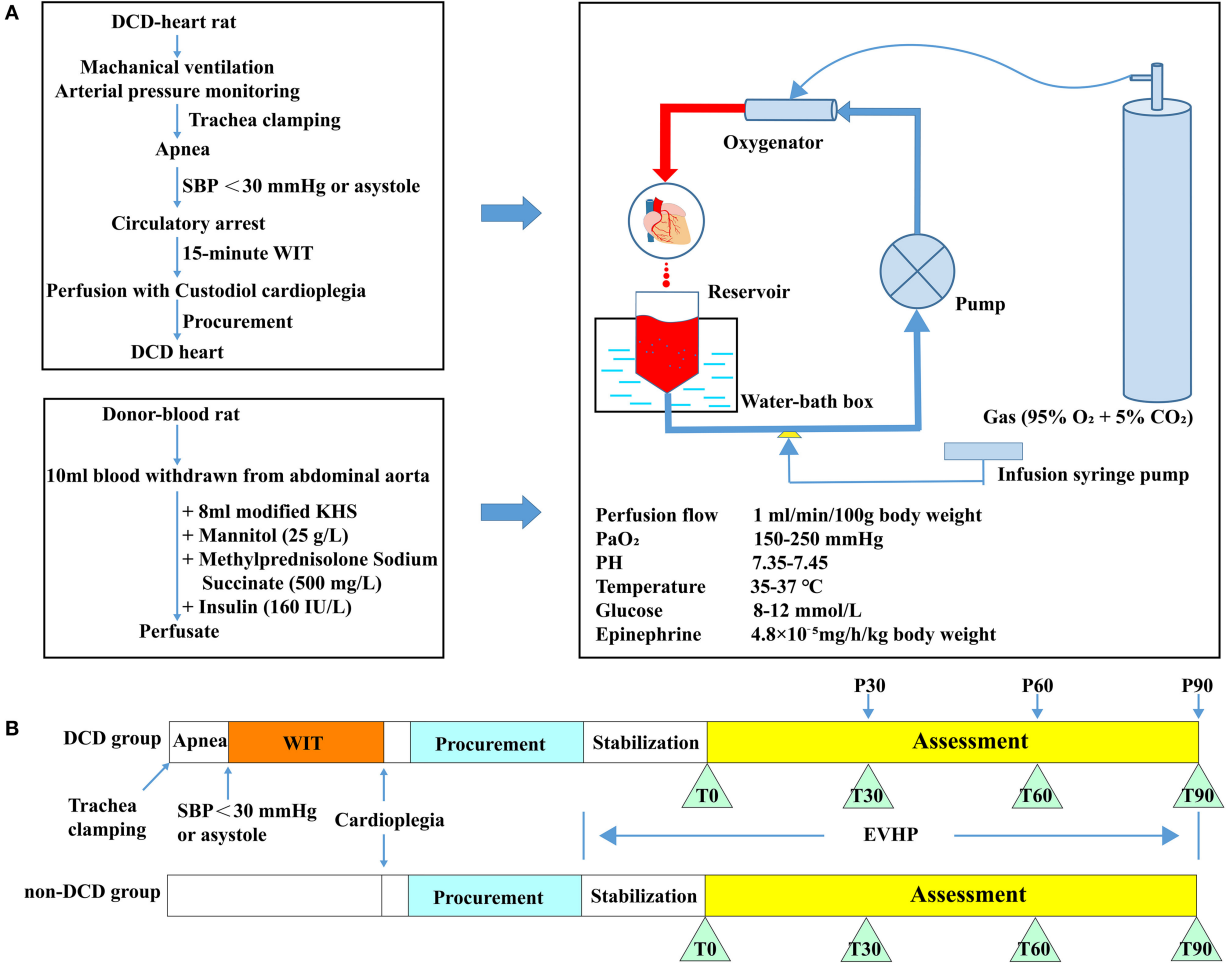
Schematic shows an overview of the protocol of the DCD model combined with normothermic ESHP and experimental design. **(A)** Protocol of the DCD model combined with normothermic ESHP; **(B)** Experimental design. DCD, Donation after circulatory death; SBP, Systolic blood pressure; ESHP, *Ex situ* heart perfusion; WIT, Warm ischemic time; KHS, Krebs-Henseleit solution. Glucose and PH were adjusted according to the result of the blood gas analysis of perfusate.

Thirty-nine male Sprague-Dawley rats (200–300 g; 8–12-week-old) were introduced as donor-heart rats. Another 39 male Sprague-Dawley rats (300–400 g; 12–15-week-old) were introduced as blood donors for the blood-based perfusate of the normothermic ESHP system (Figure 1A). All donor hearts were harvested and preserved by the normothermic ESHP system. Functional assessment of hearts and blood gas analysis were performed during the ESHP period. At the end of perfusion, heart tissue was collected for evaluation of tissue energy state, oxidative stress, apoptosis, inflammation, myocardial infarction, and histopathology.

Donor-heart rats were divided into 2 groups: (1) DCD group: 26 rats were subjected to the DCD procedure with 15 min of warm ischemia. Eight DCD hearts were submitted to 90-min normothermic ESHP, during which assessment of cardiac function was performed. And the results of blood gas analysis were presented. Another 18 DCD hearts were preserved by the normothermic ESHP system for 30, 60, or 90 min (P30, P60, and P90 groups, 6 rats for each group) and used for molecular biology analysis. (2) Non-DCD group: 13 non-DCD hearts were harvested from heart-beating rats and preserved by the normothermic ESHP system for 90 min. Assessment of cardiac function was performed in 8 hearts and the corresponding results of blood gas analysis were presented. Another 5 hearts were used for molecular biology analysis after 90-min normothermic ESHP (Figure 1B).

### Establishment of the Normothermic ESHP System

**Figure 2:**
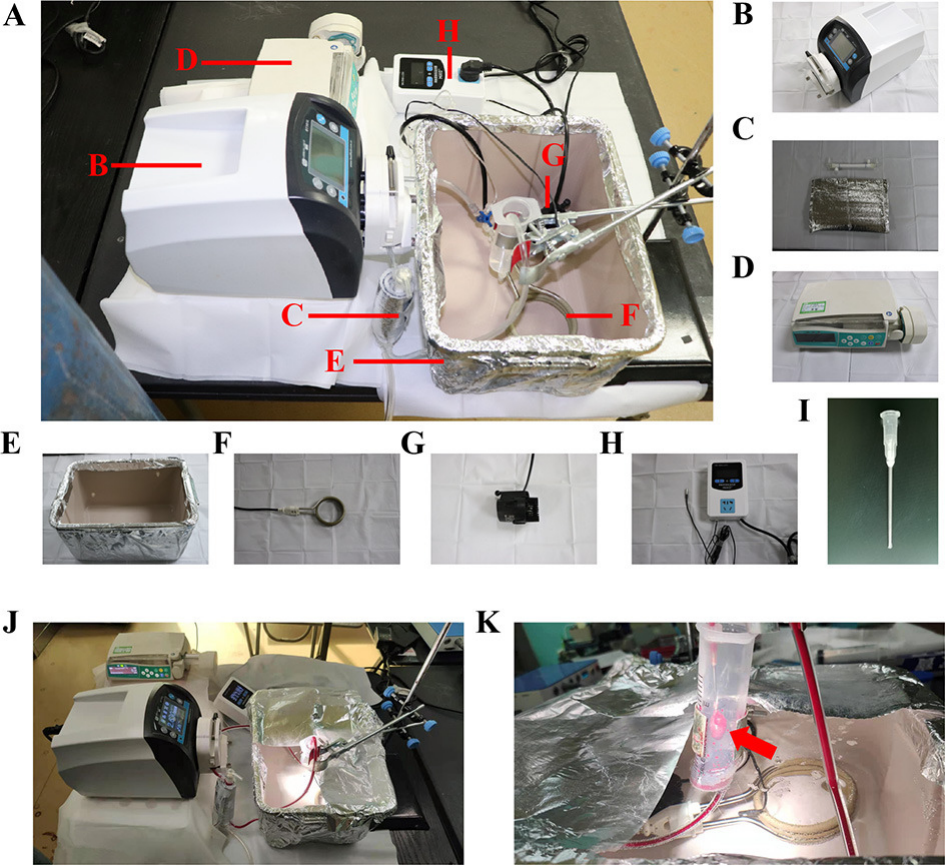
ESHP system. **(A)** Schematic figure of the ESHP system; **(B)** Micro-peristaltic pump; **(C)** Oxygenator; **(D)** Infusion syringe pump; **(E)** Water-bath box; **(F)** Heater; **(G)** Stirrer; **(H)** Temperature-controlling switch; **(I)** Aortic catheter; **(J)** Working ESHP system; **(K)** Beating heart (Red arrow). ESHP, *Ex situ* heart perfusion.

The normothermic ESHP system was depicted in Figure 2A. The perfusion circuit consisted of a micro-peristaltic pump (BT101L; Lead Fluid; China; Figure 2B), an oxygenator (Micro-1 Rat Oxygenator; Dongguan Kewei; China; Figure 2C), 16# Tygon tubing, a reservoir, an infusion syringe pump (Perfusor-space; B.Braun; Germany; Figure 2D) for the administration of epinephrine (4.8 × 10^−5^ mg/h/kg body weight), and a home-made water-bath box (Figure 2E) containing water, heater (Figure 2F), stirrer (Figure 2G) and temperature-controlling switch (Figure 2H). The oxygenator was gassed with a humidified gas mixture containing 95% O_2_/5% CO_2_. The perfusion circuit was primed with blood-based perfusate, which comprised of 10 ml blood from the donor-blood rat and 8 ml modified Krebs-Henseleit solution (10 mM glucose, 117 mM NaCl, 4.5 mM KCl, 25 mM NaHCO_3_, 1.2 mM NaH_2_PO_4_, 2 mM CaCl_2_, 0.512 mM MgCl_2_) and supplemented with mannitol (25 g/L), methylprednisolone sodium succinate (500 mg/L; Pfizer; Belgium; Switzerland), and insulin (160 IU/L; Novo Nordisk; Denmark) (Table 1). The reservoir was partially immersed in the water of the water-bath box and the isolated heart was placed below the horizontal plane of the water. The membrane oxygenator was wrapped by a thermal insulation bag and the temperature of water in the box was set at 41–42°C to maintain the temperature of the inflow and isolated heart at 35–37°C.

**Table 1:** The components of perfusate in the ESHP system.

**Components**		**Volume/concentration**
	Blood (ml)	8
Modified KHS (ml)	10 mmol/L Glucose	10
	117 mmol/L NaCl	
	4.5 mmol/L KCl	
	25 mmol/L NaHCO_3_	
	1.2 mmol/L NaH_2_PO_4_	
	2 mmol/L CaCl_2_	
	0.512 mmol/L MgCl_2_	
Methylprednisolone sodium succinate (mg/L)		500
	Mannitol (g/L)	25
	Insulin (IU/L)	160

*ESHP, Ex situ heart perfusion; DCD, Donation after circulatory death; KHS, Krebs-Henseleit solution*.

### Operative Procedure

#### Anesthesia

Sprague-Dawley rats were anesthetized using 3% isoflurane in an inhalation chamber. Once the rat was unresponsive, an intraperitoneal injection of ketamine (75 mg/kg) and xylazine (5 mg/kg) was performed to maintain the anesthetic effect for the rest of the procedure. Pedal reflex was used to determine adequate anesthetic depth before experiments. The rats were placed on a heating pad to maintain the body temperature.

#### Harvest of Blood From the Donor-Blood Rat

A midline incision in the abdomen of the donor-blood rat was made to access the abdominal artery. An 18 G, 2-inch I.V. catheter with a 3-way stop cock was cannulated into the abdominal artery. Then a 20 ml syringe containing 1,250 IU heparin was connected to the catheter to withdraw 10 ml blood. The blood was heparinized by the heparin inside and mixed with 8 ml modified Krebs-Henseleit solution. Then mannitol, methylprednisolone sodium succinate, and insulin were added into the perfusate. The blood-based perfusate was slowly added into the perfusion circuit to be oxygenated for 15 min (PaO_2_ at 150–250 mmHg).

#### Procurement of Heart From the Donor-Heart Rat

A midline incision in the neck of the donor-heart rat was made to access the trachea. Then the rat was intubated with a 16 G, 2-inch I.V. catheter after tracheotomy and mechanically ventilated with air at 60 breaths per min, with tidal volume ranging from 8 to 10 ml/kg body weight. A 22 G, 1-inch I.V. catheter was cannulated into the right carotid artery. The catheter was connected to a 3-way stop cock, allowing the injection of heparin, the connection of pressure transducer for real-time blood pressure (perfusion pressure later) monitoring, and the delivery of Custodiol cardioplegic solution (Dr. Franz Köhler, Chemie GmbH, Bensheim, Germany). Heparinization of the donor-heart rat was achieved by the slow injection of heparin (2,000 IU/kg body weight) through the right carotid artery.

Rats in the DCD group were subjected to the following DCD procedures. To induce the circulatory death, the rat was extubated and the trachea was clamped by a mosquito forceps to initiate the apnea. The circulatory death was declared when systolic pressure below 30 mmHg or asystole was observed (14, 17). Then 15-min warm ischemic time (WIT, equivalent to the hands-off period) was allowed to elapse. At the end of the 15-min WIT, the sternotomy was performed and the aortic arch between the brachiocephalic trunk and left common carotid artery was clamped. After cutting the inferior vena cava and opening the left atrium, the DCD heart was immersed with ice and perfused with 20 ml cold Custodiol cardioplegic solution at a constant pressure of 60–80 mmHg for 3–5 min *via* the right carotid artery. Subsequently, aorta distal to the left subclavian artery and pulmonary artery were cut and the arrested heart was excised and submerged into ice-cold Krebs-Henseleit solution. The harvested heart was cannulated with a homemade 14 G aortic catheter (Figure 2I) through the aorta carefully without damaging the aortic valve leaflets. Then removed the air from the aorta and catheter by dripping cold modified Krebs-Henseleit solution and tightly fixed the aorta to the cannula using a 2-0 silk suture to make sure no leakage from the aorta was detected. Before the initiation of ESHP, the donor heart was immersed in the hypothermic modified Krebs-Henseleit solution. Rats in the non-DCD group were treated similarly, but not submitted to 15-min warm ischemia.

#### The Operation of ESHP

After 15-min oxygenation for the perfusate, the donor heart was connected to the ESHP system *via* the aortic cannula. The ESHP started with an initial perfusion flow rate of 2 ml/min, and slowly reached the target perfusion flow rate (1 ml/min/100 g body weight) within 10 min (Figures 2J,K). Once the target perfusion flow rate was reached, the administration of epinephrine (4.8 × 10^−5^ mg/h/kg body weight) was initiated and continued *via* an infusion syringe pump throughout the perfusion. The initiation of ESHP was considered to be the beginning of the stabilization period (Figure 1B). During the ESHP, PaO_2_ was maintained at 150–250 mmHg and the temperature of the isolated heart at 35–37°C. PH (7.35–7.45) and glucose level (8–12 mmol/L) of perfusate were adjusted with 50% glucose solution and 5% sodium bicarbonate solution according to the blood gas analyses.

### Cardiac Functional Assessment and Blood Gas Analysis During ESHP

At the end of a 15-min stabilization period, the latex balloon connected to a pressure sensor was inserted into the left ventricle through the left atrium. The assessment phase started at end of the 15-min stabilization and lasted for 90 min. The intraventricular pressure measurement recording was performed by slowly filling the balloon with 0.15 ml saline at the beginning of the assessment phase (T0) and then every 30 min (T30, T60, T90). The cardiac functional parameters of the donor heart during ESHP included developed pressure (DP, systolic blood pressure minus diastolic blood pressure), heart rate (HR), dP/dt_max_ (maximum rate of rise of left ventricular pressure), and dP/dt_min_ (maximum rate of pressure decline). Besides, the blood gas analysis of perfusate was performed at T0, T30, T60, and T90 using a blood gas analysis instrument (Gem Premier 3,500; Instrumentation Laboratory; USA). In particular, the blood gas analysis only presents the concentration of ionized calcium, and the hematocrit below 15% and hemoglobin below 51 g/L are presented as missing values. There is explicit relation between the values of hematocrit and hemoglobin in the blood gas analysis, which means if hematocrit is below 15%, the hemoglobin will be below 51 g/L.

### Sample Collection

At the end of the experiment, four ventricular slices (1- to 2-mm thick) were collected serially along the long axis. The first piece of myocardial tissue from the apex was used for the measurement of adenosine triphosphate (ATP) level, the second one for analyzing superoxide dismutase (SOD) activity, and the level of glutathione (GSH) and malonaldehyde (MDA), the third one for histologic and immunohistochemical analysis, and the fourth one for the evaluation of myocardial infarct size.

### Analysis

#### Tissue Energy State

Myocardial tissue was weighed and diluted 1:9 with boiled double distilled water to prepare tissue homogenate and then centrifugated for supernatant collection. Tissue ATP levels were measured by phosphomolybdic acid colorimetry using a bioluminescence kit (A095-1-1, Nanjing Jiancheng Bioengineering Institute, China). The analysis was performed using an automatic microplate reader (CLARIOstar, BMG Labtech, Germany).

#### Oxidative Stress

Myocardial tissue was weighed and diluted 1:9 with normal saline to prepare tissue homogenate. After centrifugation, the supernatant was collected for examination. The SOD activity and the levels of MDA and GSH in the supernatant were determined by colorimetric 5,5′-dithio-bis-(2-nitrobenzoic acid)-based method, thiobarbituric acid reactive substance assay, and water-soluble tetrazolium-1 method respectively (A001-3-2, A003-1-2, A006-2-1, Nanjing Jiancheng Bioengineering Institute, China). The analysis was performed using an automatic microplate reader (CLARIOstar, BMG LAGTECH, Germany). The expression of 4-Hydroxynonenal (HNE), an indicator of oxidative stress, was determined by immunohistochemical analysis as described below.

#### Inflammatory Response

As indicators of the inflammatory response, the expression of interleukin-6 (IL-6) and tumor necrosis factor-α (TNF-α) were measured by immunohistochemistry as described below.

#### Immunohistochemistry

Myocardial tissue slices were fixed in paraformaldehyde solution (4%), embedded in paraffin, and cut to 4-μm-thick sections. The immunoreactivity to IL-6 (1:500, Abcam, ab9324, USA), TNF-α (1:200, Abcam, ab109322, USA), HNE (1:1,000, Abcam, ab46545, USA) was assessed. The antigen-antibody reaction was visualized by diaminobenzidine reaction, and randomly selected fields from each slice were recorded under a conventional light microscope in a blinded fashion. Image analysis was performed using Image-Pro Plus software (Media Cybernetics, USA). The evaluation was carried out in four random and non-verlapping fields of the heart tissue, and the average value was calculated for each animal. The expression of IL-6, TNF-α, and HNE was determined by counting integrated optical density (IOD).

#### Histology

The paraffin blocks described above were additionally cut into 4-μm-thick slices, and stained with hematoxylin-eosin. Four random and non-overlapping visual fields for each heart slice were selected under a light microscope and the average histopathological score of four different fields was calculated for each sample in a blinded manner. We evaluated the pathology of each heart slice by scoring according to the grades 0–4: 0: Nil, (1) Minimum (Focal myocytes damage), (2) Mild (Occasionally disordered myocardial fibers with multifocal myofibrillar degeneration and inflammatory process), (3) Moderate (Diffuse inflammation and/or comprehensive myofibrillar degeneration with wave-shaped myocardial fibers and shed nuclei), (4) Severe (Diffuse inflammatory process with myocardial necrosis: The nuclei shrink and the cells are severely damaged) (19). Cell swelling was evaluated by calculating the mean area per cell with Image J (National Institute of Mental Health).

#### Apoptosis

Four-micrometer-thick slices from the paraffin blocks described above were used for Terminal deoxynucleotidyl transferase dUTP nick end labeling (TUNEL) staining to detect DNA-strand breaks of the donor hearts and the TUNEL staining was performed as previously described (20). The number of TUNEL-positive cells was counted under a fluorescence microscope and the final results were expressed as the ratio of 4′,6-diamidino-2-phenylindole (DAPI)-TUNEL double-labeled nuclei to the total number of nuclei stained with DAPI.

#### Evaluation of Infarct Size

Slices for 2,3,5-triphenyl-tetrazolium chloride (TTC) staining were rapidly excised at the end of the experiment. Then slices were stained with 2% TTC (Sigma-Aldrich, Shanghai, China) in phosphate-buffered saline (pH 7.4, Life Technologies, Grand Island, NY, USA), for 30 min at 37°C to demarcate the viable and non-viable myocardium. The percentage of the infarcted area was evaluated in a blinded manner by using Image J.

### Statistical Analysis

The results were reported in the median and interquartile range. Comparison between groups of P30, P60, and P90 was analyzed by Kruskal-Wallis test and then Dunn's multiple comparisons test. The data of the non-DCD group was only compared to those exposed to 90-min ESHP in the DCD group by Mann-Whitney-test. Intraventricular pressure measurement recordings and results of blood gas analysis between DCD and non-DCD groups were compared by Mann-Whitney-test according to different timepoints. A value of *p* < 0.05 was considered statistically significant. GraphPad Prism 8.3 software (GraphPad Sofware, Inc., San Diego, CA, USA) was used to perform statistical analysis.

## Results

**Figure 3:**
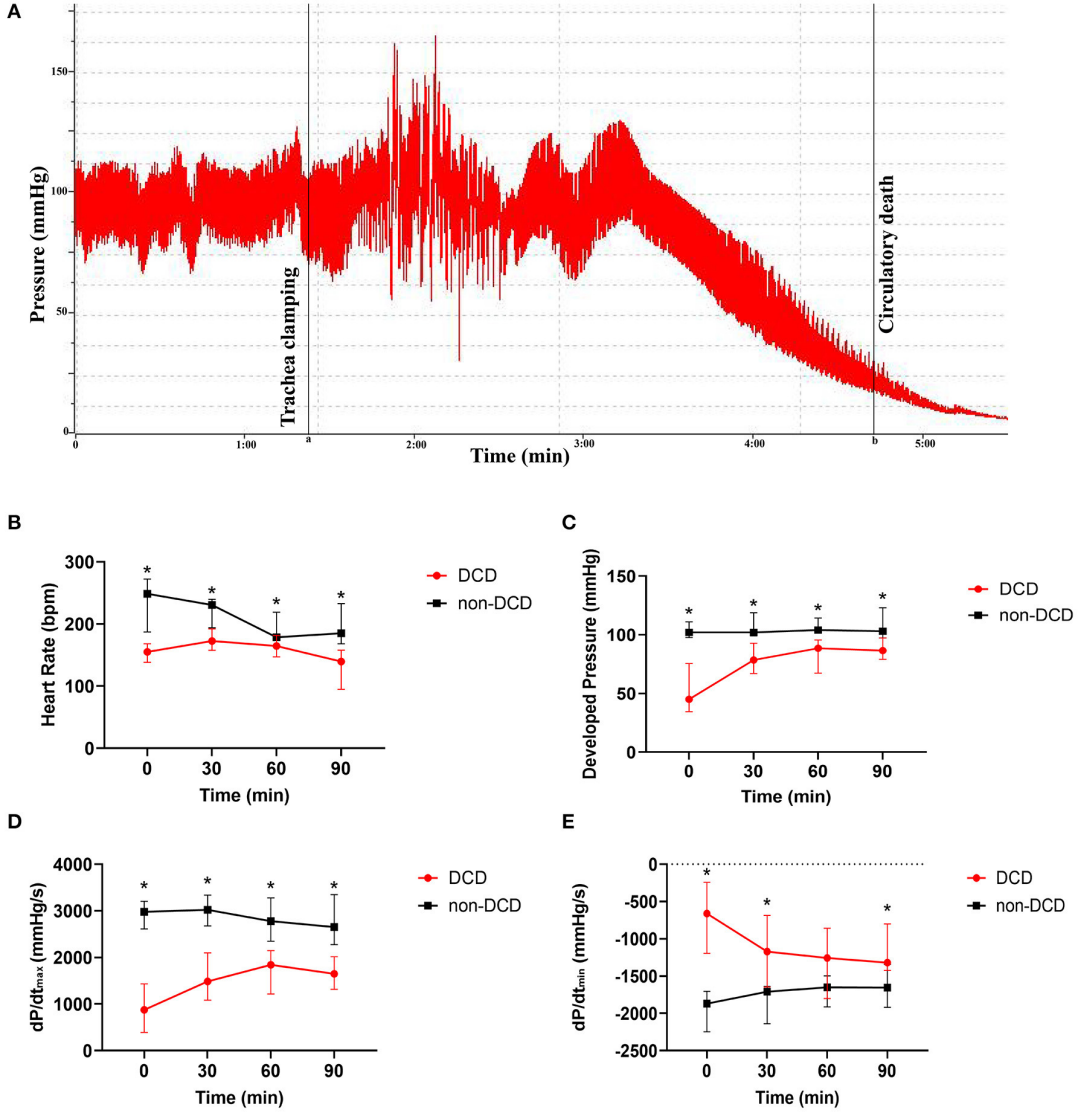
Cardiac functional assessment of the donor heart during ESHP. **(A)** The typical development process of intracarotid blood pressure of DCD-heart rat following extubation; Line a: the initiation of asphyxiation; Line b: the declaration of circulatory death; **(B)** Heart Rate; **(C)** Developed pressure; **(D)** dP/dt_max_; **(E)** dP/dt_min_. Data represented as median and interquartile range. *n* = 8 for each group. DCD, Donation after circulatory death; ESHP, *Ex situ* heart perfusion; dP/dt_max_, Maximum rate of rise of left ventricular pressure; dP/dt_min_, Maximum rate of pressure decline. *p < 0.05 vs. non-DCD group.

The blood pressure of DCD-heart rats rapidly decreased following apnea (Figure 3A). The expected time to circulatory death was 225.5 s (interquartile range, 188.8–248.3). The time between cardioplegia perfusion and the initiation of ESHP was 15 min (interquartile range, 12–22) in the DCD group and 12 in the non-DCD group (interquartile range, 8–16.5). Aortic intramural hematoma caused by mishandling happened in two hearts in the DCD group, which stopped beating within 30–60 min after reperfusion. One DCD heart failed to resume sinus rhythm during the whole period of ESHP, and the others resumed sinus rhythm within 5 min after reperfusion. Consequently, another three experiments were performed.

### Cardiac Functional Assessment of the Donor Heart During ESHP

During the assessment phase of ESHP, the HR of DCD hearts increased from T0 to T30, and slowly decreased from T30 to T90, while the DP and dP/dt_max_ in the DCD group rapidly increased from T0 to T30 and maintained a stable level from T30 to T90. Besides, the dP/dt_min_ of DCD hearts decreased obviously in T30 and remained stable in the subsequent 60 min. In the non-DCD group, the DP and dP/dt_min_ did not alter obviously during perfusion, while the HR and dP/dt_max_ slightly decreased in T60 and T90. Moreover, the HR, DP, and dP/dt_max_ of non-DCD hearts were significantly higher than that of DCD hearts during the whole ESHP period (Figures 3B–D). And lower dP/dt_min_ was observed in the non-DCD group compared to the DCD group. The difference of dP/dt_min_ between DCD and non-DCD groups was significant in T0, T30, and T90 (Figure 3E).

### Blood Gas Analysis of the Perfusate During ESHP

During ESHP, the blood gas analysis of perfusate was performed in T0, T30, T60, and T90. Generally, the level of Na^+^, K^+^, and lactate rose, while Ca^2+^ in the perfusate decreased during ESHP in both DCD and non-DCD groups. Moreover, both groups showed that the level of hemoglobin and hematocrit in the perfusate slightly increased from T0 to T60 and dropped in T90. The K^+^ concentration in the DCD group was significantly higher than that in the non-DCD group in T30, T60, and T90, while the lactate level was significantly lower in T0, T30, and T60 in the DCD group compared to the non-DCD group. The differences of Na^+^, hemoglobin, and hematocrit between DCD and non-DCD groups in different timepoints were not statistically significant (Table 2).

**Table 2:** The blood gas analysis of the perfusate during ESHP.

**Variables**	**DCD group**				**Non-DCD group**			
	**T0**	**T30**	**T60**	**T90**	**T0**	**T30**	**T60**	**T90**
Na^+^ (mmol/L)	134.0 (131.3–136.0)	136.5 (131.5–138.8)	136.0 (135.0–138.0)	136.5 (133.8–143.8)	135.5 (130.5–141.0)	138.5 (131.8–145.5)	141.0 (135.0–149.3)	143.0 (139.3–150.8)
K^+^ (mmol/L)	4.8 (4.6–5.4)	5.5 (5.2–5.9)^*^	6.0 (5.6–7.3)^*^	6.4 (6.0–9.3)^*^	4.3 (4.1–5.1)	4.4 (4.2–5.4)	4.5 (4.1–5.6)	5.1 (4.2–6.3)
Ca^2+, a^ (mmol/L)	0.96 (0.80–1.01)	0.94 (0.74–1.00)	0.93 (0.69–0.99)	0.92 (0.61–0.96)	0.94 (0.82–1.03)	0.88 (0.75–1.01)	0.84 (0.66–0.97)	0.80 (0.60–0.96)
Lactate (mmol/L)	3.7 (3.4–4.1)^*^	5.3 (4.8–5.5)^*^	6.9 (5.8–7.5)^*^	8.7 (6.5–9.9)	4.5 (4.3–5.1)	6.9 (6.5–7.7)	8.3 (7.4–9.6)	10.5 (8.3–11.4)
Hemoglobin^b^ (g/L)	65.0 (63.0–69.5)	65.0 (63.0–66.5)	68.0 (56.0–71.5)	58.0 (54.5–69.5)	66.5 (59.8–78.5)	68.0 (60.5–80.8)	71.0 (63.5–79.3)	68 (56.8–79.3)
HCT^b^ (%)	19.0 (18.5–20.5)	19.0 (18.5–19.5)	20.0 (16.5–21.0)	17.0 (16.0–20.5)	19.5 (17.5–23.0)	20.0 (17.8–23.8)	21.0 (18.8–23.3)	20.0 (16.8–23.3)

*Data represented as median and interquartile range. n = 8 and n = 6 for DCD and non-DCD groups respectively. HCT, Hematocrit*.

a *The content of calcium listed in this table represents the ionized calcium concentration*.

b *The hematocrit below 15% and hemoglobin below 51 g/L are presented as missing values in blood gas analysis. The DCD group had 1 missing value of hemoglobin and HCT in T60 and 3 in T90, while the non-DCD group had 1 missing value of hemoglobin and HCT in T0, T30, and T60 respectively. If there was a missing value in the repeated measurement, the corresponding values in other timepoints were excluded (n = 5 remained in the DCD group, n = 4 remained in the non-DCD group)*.

* *p < 0.05 vs. non-DCD group*.

### Tissue Energy State of the Donor Heart During ESHP

**Figure 4:**
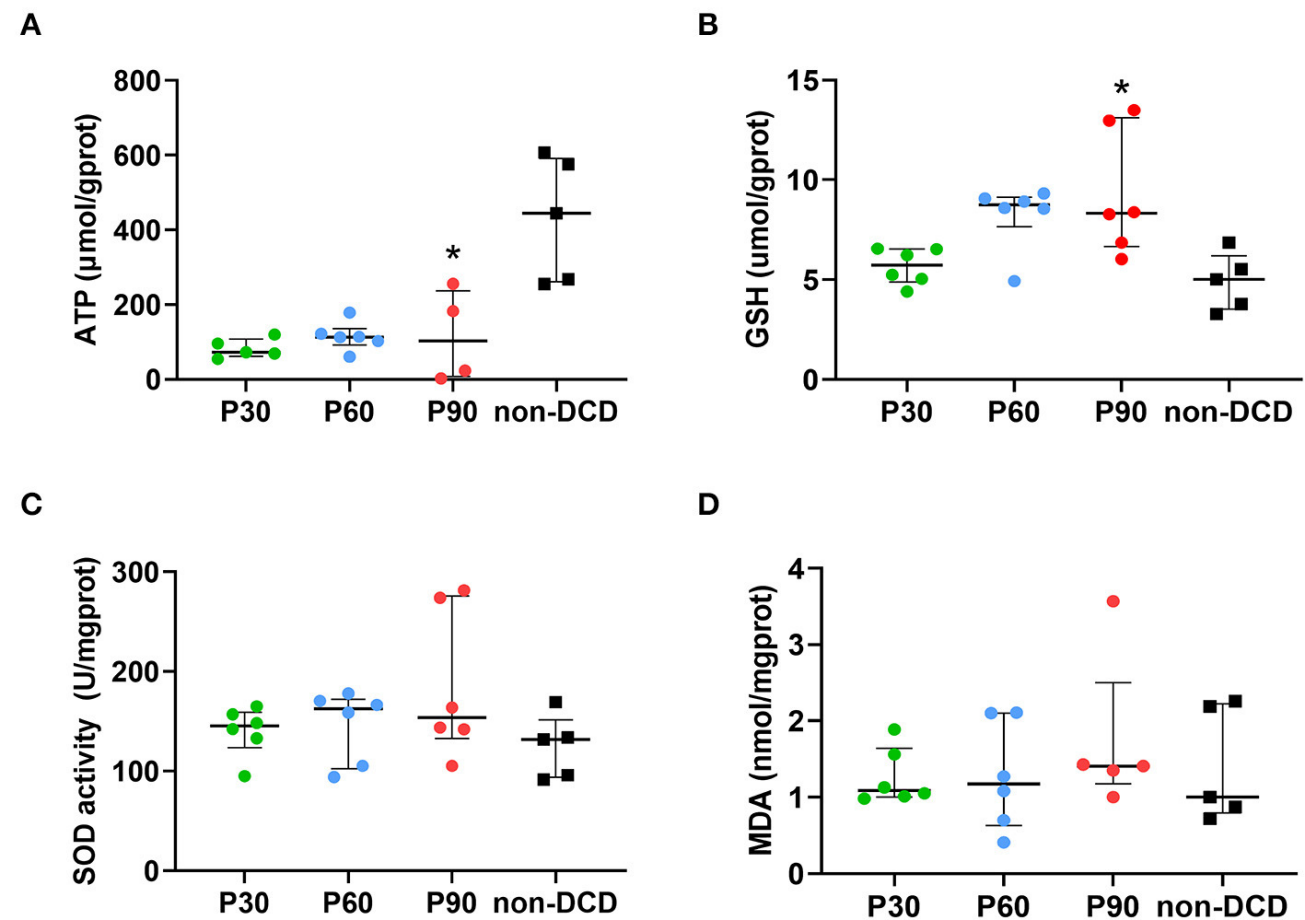
Tissue content of ATP, GSH, MDA, and SOD activity in the donor heart during ESHP. **(A)** ATP. *n* = 5 for P30 group and non-DCD group, *n* = 6 for P60 group, *n* = 4 for P90 group; **(B)** GSH. *n* = 6 for P30, P60, and P90 groups, *n* = 5 for non-DCD group; **(C)** SOD activity. *n* = 6 for P30, P60, and P90 groups, *n* = 5 for non-DCD group; **(D)** MDA. *n* = 6 for P30 and P60 groups; *n* = 5 for P90 group and non-DCD group. Data represented as median and interquartile range. ATP, Adenosine triphosphate; DCD, Donation after circulatory death; ESHP, *Ex situ* heart perfusion; GSH, Glutathione; MDA, Malonaldehyde; SOD, Superoxide dismutase. *p < 0.05 vs. non-DCD group.

Three values below zero of ATP were excluded (2 samples in the P90 group, 1 sample in the P30 group). Figure 4A showed that no significant difference was found in the ATP content of DCD hearts among the groups of P30, P60, and P90. The non-DCD hearts showed a significantly higher level of ATP compared to DCD hearts at the end of 90-min ESHP.

### Oxidative Stress of the Donor Heart During ESHP

**Figure 5:**
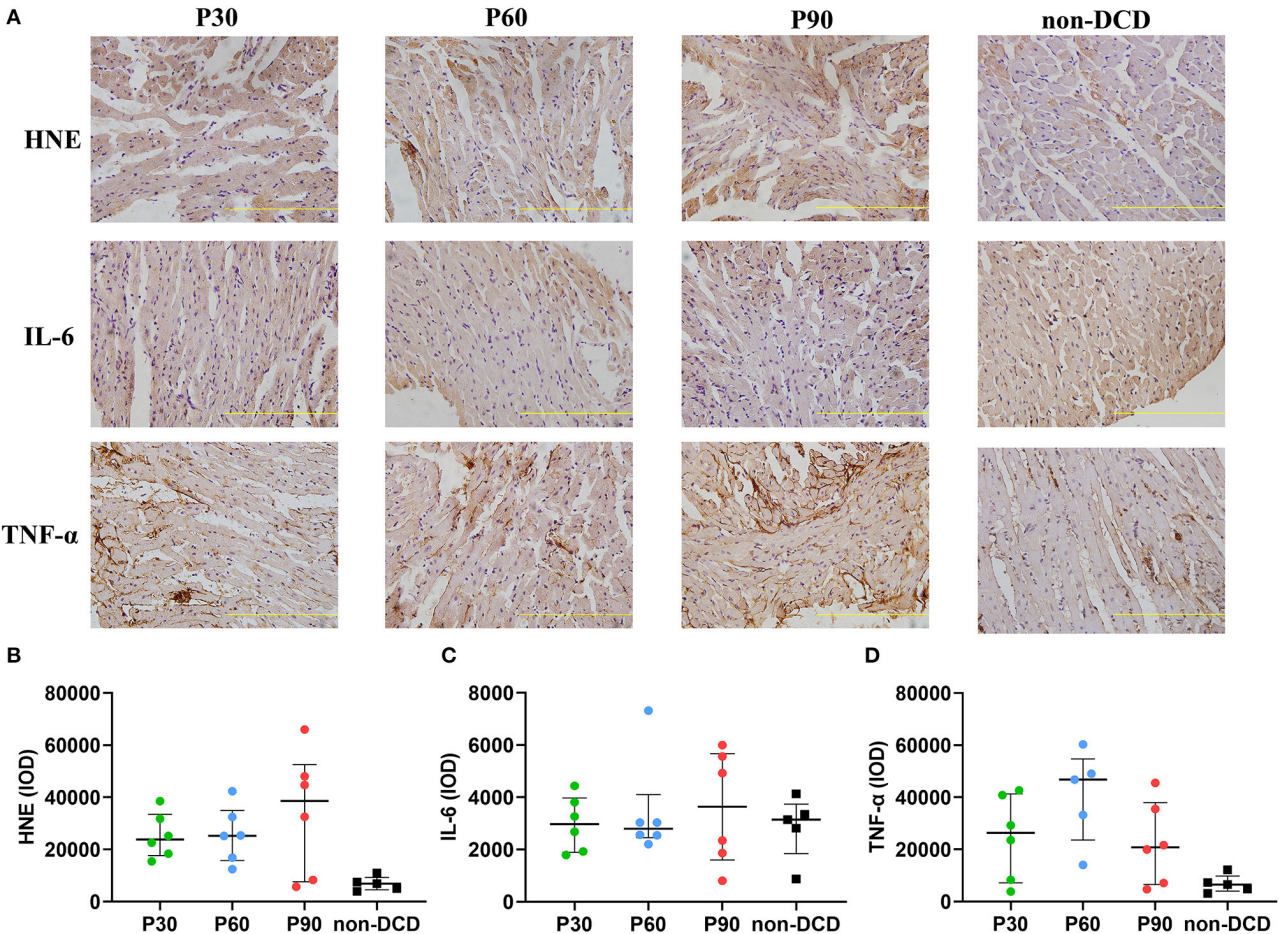
The expression of HNE, IL-6, and TNF-α in the donor heart during ESHP. **(A)** Representative photomicrographs of Immunohistochemistry for donor hearts. Bar scale: 100 μm; **(B)** Mean HNE IOD. *n* = 6 for P30, P60, and P90 group, *n* = 5 for non-DCD group; **(C)** Mean IL-6 IOD. *n* = 6 for P30, P60, and P90 groups, *n* = 5 for non-DCD group; **(D)** Mean TNF-α IOD. *n* = 6 for P30 and P90 groups; *n* = 5 for P60 group and non-DCD group. Data represented as median and interquartile range. DCD, Donation after circulatory death; ESHP, *Ex situ* heart perfusion; HNE, 4-Hydroxynonenal; IL-6, Interleukin-6; IOD, Integrated optical density; TNF-α, Tumor necrosis factor-α.

In DCD hearts, the level of GSH in the P30 group was lower than that in the P60 and P90 groups without reaching a significant difference. The GSH level was significantly higher in DCD hearts after 90-min ESHP compared to non-DCD hearts (Figure 4B). No significant difference was observed regarding the SOD activity and the level of MDA in the DCD hearts among groups of P30, P60, and P90. Both the SOD activity and MDA level in the DCD and non-DCD hearts after 90-min ESHP were not significantly different (Figures 4C,D). As shown in Figures 5A,B, the expression of HNE in DCD hearts was not significantly different among groups of P30, P60, and P90. At the end of 90-min ESHP, the HNE expression in the DCD hearts was higher than non-DCD hearts without reaching a significant difference.

### Inflammatory Response of the Donor Heart During ESHP

The expression of IL-6 and TNF-α in heart tissue was measured by immunohistochemistry to evaluate the inflammatory response during ESHP. The levels of IL-6 and TNF-α were not significantly different in DCD hearts among the groups of P30, P60, and P90. After 90-min ESHP, the IL-6 expression was similar in DCD and non-DCD hearts, while the TNF-α expression in non-DCD hearts was lower than that in DCD hearts without reaching a significant difference (Figures 5A,C,D).

### Histopathological Assessment of the Donor Heart During ESHP

**Figure 6:**
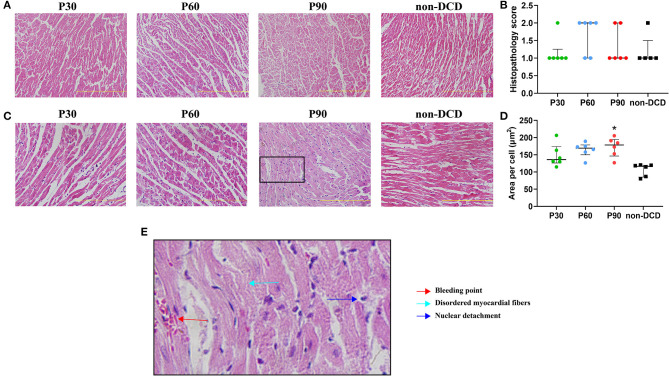
Histopathological changes of donor heart during ESHP. **(A)** Representative photomicrographs of hematoxylin and eosin staining for donor hearts under magnification of 20 (Scale length: 200 μm); **(B)** Histopathology score. *n* = 6 for P30, P60, and P90 groups, *n* = 5 for non-DCD group; **(C)** Representative photomicrographs of hematoxylin and eosin staining for donor hearts under magnification of 40 (Scale length: 100 μm); **(D)** Mean area per cell. *n* = 6 for P30, P60, and P90 groups, *n* = 5 for non-DCD group; **(E)** Schematic diagram of damaged myocardium in hematoxylin and eosin staining. Data represented as median and interquartile range. DCD, Donation after circulatory death; ESHP, *Ex situ* heart perfusion. *p < 0.05 vs. non-DCD group.

DCD hearts preserved by the ESHP showed few focal myocytes damage, occasionally disordered myocardial fibers, and bleeding, but no indication of acute inflammation and necrosis. The histopathological score was 1 in the P30 group (interquartile range, 1–1.25), 2 in the P60 group (interquartile range, 1–2), 1 in the P90 group (interquartile range, 1–2), and 1 in the non-DCD group (interquartile range, 1–1.5). Moreover, no significant difference in histopathological score and the mean area per cell (the indicator of cell swelling) of heart tissue was observed among the groups of P30, P60, and P90. DCD and non-DCD groups had similar histopathological scores after 90 min of ESHP. The mean area per cell was significantly lower in the non-DCD hearts compared to the DCD hearts at the end of 90-min ESHP, indicating less myocardial edema in the non-DCD hearts after perfusion (Figure 6).

### Apoptosis of the Donor Heart During ESHP

**Figure 7:**
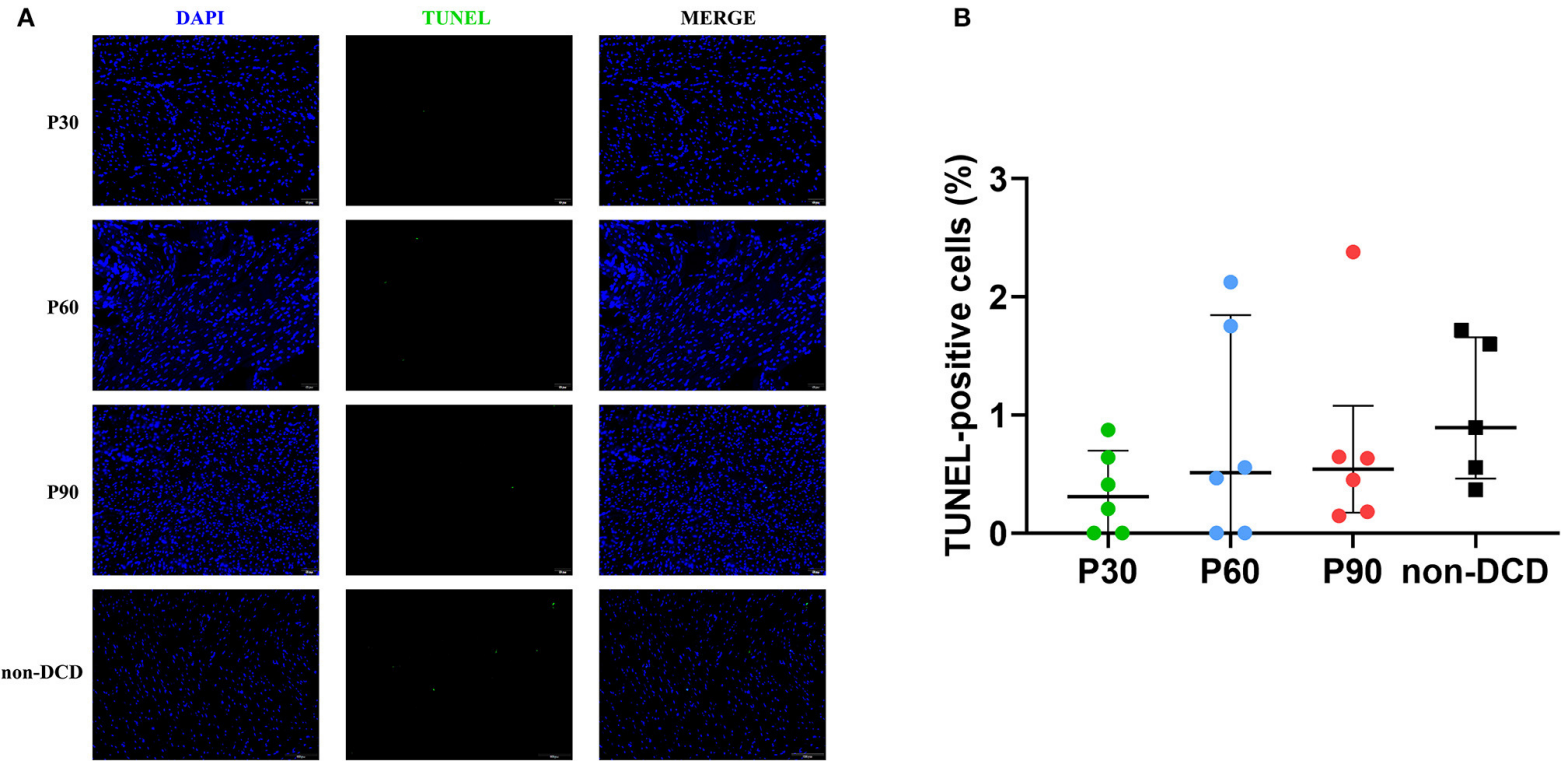
Apoptosis evaluation of the donor heart during ESHP. **(A)** Representative photomicrographs of myocardial tissue stained with DAPI (blue), nuclei with fragmented DNA shown by TUNEL staining, and merged image (magnification of 20; scale length: 50 μm); **(B)** Quantification of TUNEL-positive cells (as a percentage). *n* = 6 for P30, P60, and P90 groups, *n* = 5 for non-DCD group. Data represented as median and interquartile range. DAPI: 4′,6-diamino-2-phenylindole (DAPI, blue); DCD, Donation after circulatory death; ESHP, *Ex situ* heart perfusion; TUNEL, terminal deoxynucleotidyl transferase-mediated dUTP nick end-labeling.

The myocardial apoptosis was not obvious in the DCD hearts during ESHP and not significantly different among the groups of P30, P60, and P90. The apoptosis of myocardial cells was also moderate in the non-DCD hearts, which was not significantly different from the DCD hearts subjected to 90 min of ESHP (Figure 7). The negative and positive control for the TUNEL staining of myocardial tissue were presented in Supplementary Figure 1.

### Infarct Size Evaluation of the Donor Heart During ESHP

**Figure 8:**
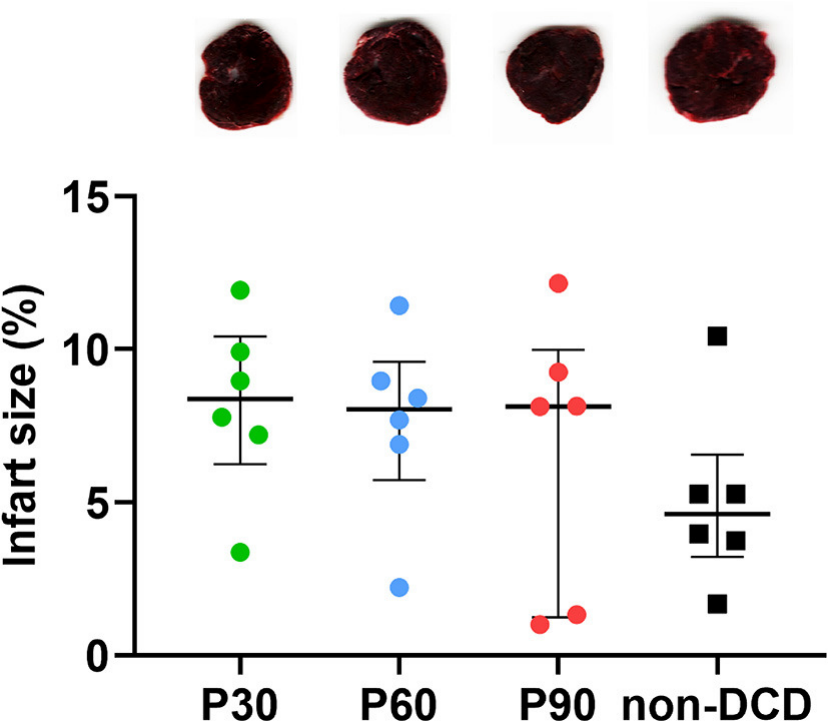
Evaluation of infarct size for the donor heart during ESHP. Representative photomicrographs of TTC staining for the donor hearts during ESHP. Brick red: Live tissue; Pale tissue: Dead tissue. The lower panel showed the quantification of infarct size for the DCD hearts. *n* = 6 for P30, P60, and P90 groups, *n* = 5 for non-DCD group. Data represented as median and interquartile range. DCD, Donation after circulatory death; ESHP, *Ex situ* heart perfusion; TTC, 2,3,5-Triphenyl-tetrazolium chloride.

As depicted in Figure 8, no obvious myocardial infarction was observed in both DCD and non-DCD hearts. Moreover, no significant difference was found in the infarct size of DCD hearts among the groups of P30, P60, and P90. The infarct size of the non-DCD hearts was insignificantly lower than the DCD hearts after 90 min of ESHP.

## Discussion

We established a clinically relevant and reproducible model of normothermic ESHP for DCD hearts in rats. Our data demonstrated that the functional performance of DCD hearts was compromised at the beginning of ESHP and recovered over time, while non-DCD hearts presented better cardiac function during ESHP. Higher potassium concentration during ESHP was observed in the DCD group, which might be attributed to the release of potassium from myocardial cells injured by warm ischemia. Moreover, DCD hearts submitted to different durations of ESHP showed mild histopathological alterations, but the DCD hearts had more evident cell swelling compared to non-DCD hearts after 90-min ESHP. No obvious myocardial infarction was observed in the DCD hearts among different durations of ESHP and the non-DCD hearts after 90-min ESHP. The alteration of DCD hearts in oxidative stress, apoptosis, tissue energy state, histopathological changes, cell swelling, infarction, and inflammatory response didn't differ among different durations of ESHP. At the end of 90-min ESHP, the DCD and non-DCD hearts presented similarly in the level of apoptosis, infarction, and histopathological changes. The hearts in the DCD group had lower energy storage, indicating mitochondrial dysfunction and compromised aerobic metabolism in DCD hearts. Besides, the expression of HNE and TNF-α appeared to be higher in the DCD hearts, which might be related to more serious inflammatory response and oxidative stress in DCD hearts. On the contrary, the DCD hearts showed higher GSH levels than non-DCD hearts. GSH is an important reducing agent and the higher level of GSH in the DCD group might be the compensatory response to warm ischemia.

Currently, HTx with DCD hearts has received much attention over the past 15 years (21, 22) and is regarded as a promising strategy to expand the donor pool (23). However, the severe IRI due to the inherent warm ischemia limits the adoption of DCD hearts in HTx (1, 2, 5). Normothermic ESHP has become a novel and ground-breaking strategy which has been applied for DCD heart preservation in the clinical setting and provides a unique chance to recondition the DCD hearts. Indeed, TransMedics® Organ Care System (TransMedics, USA) is commercially available for DCD heart preservation and transportation, which generally consists of a pump, membrane oxygenator, heat exchanger, blood reservoir, and perfusion solution with donor blood as the main component (9). Therefore, the establishment of an animal model of normothermic ESHP is urgently necessary to assess the efficacy of post-conditioning cardioprotective pharmacological agents delivered into the perfusion circuit to recondition DCD hearts (24). As porcine models are expensive and labor-intensive, a simple rat model of cardiac DCD combined with ESHP is more convenient and economical for preliminary studies of reconditioning DCD hearts with cardioprotective pharmacological agents through the ESHP system.

Rat models of cardiac DCD combined with ESHP have been reported in several studies, in most of which the normothermic ESHP was achieved by the langendorff system (14–18). The langendorff system was established in the 1890s by Oscar Langendorff for isolated heart preparation (25). Langendorff system is primed with crystalloid solution and then perfuses the hearts at 37°C at constant pressure or constant flow. Oxygen is provided by bubbling the priming solution with a mixture of 95% O_2_/5% CO_2_. However, because the lack of hemoglobin in crystalloid solution leads to low oxygen-carrying capacity and low oxygen dissociation rate in cardiac tissue, artificially high coronary flow is always necessary for the isolated hearts (25, 26). In these studies (14–18), the authors perfused DCD hearts with Krebs-Henseleit solution at 37°C with constant perfusion pressure within 60–70 mmHg for 60 or 120 min. Since global warm ischemia can reduce the coronary flow with increased vascular resistance (27), an insufficient supply of oxygen and unmet metabolic demand of DCD hearts might exist in these models, as indicated by the reduced coronary flow (15) and the obvious myocardial infarction after ESHP (16, 17). Quader et al. suggested that crystalloid-based perfusate might not be able to maintain the basal oxygen demand of DCD hearts during normothermic ESHP and require an oxygen carrier molecule (28). Bell et al. also suggested that blood perfusion of isolated hearts was associated with better preservation of left ventricular function following IRI (26). Further studies are necessary to investigate whether the langendorff system primed with the crystalloid solution could reflect the cardioprotective effect of normothermic ESHP for DCD heart preservation.

Tolboom et al. (12, 13) have built up a clinically relevant normothermic ESHP system for the rat DCD hearts, including a jacketed perfusion chamber, a membrane oxygenator, a heat exchanger, and a peristaltic pump. The perfusion solution was autologous blood diluted 1:1 with Krebs-Henseleit solution and oxygenated using a membrane oxygenator with a mixture of 95% O_2_/5% CO_2_. The DCD hearts were perfused at a constant flow. It was a pity that the details of the establishment and management of ESHP in the studies performed by Tolboom et al. were insufficiently described.

Exsanguination and asphyxiation are two major strategies to induce circulatory death for *in situ* ischemia rat models of DCD. However, asphyxiation is preferable as DCD hearts are mainly harvested from Maastricht category III donors so far (29). Compared with exsanguination, an entire circulatory load is unavoidable for the DCD hearts during hypoxia caused by asphyxiation (30). Besides, hypoxic pulmonary vasoconstriction following asphyxiation results in right ventricular dilatation and more damage to hearts compared with exsanguination (3, 31). To induce asphyxiation, Quader et al. terminated the ventilation support following paralyzing skeletal muscles with muscle relaxants (32, 33), while Tolboom et al. chose to transect the diaphragm (12, 13). Asphyxiation can also be induced by tracheal clamping or by median sternotomy and opening the pleurae (14, 16–18). Aceros et al. and Kearns et al. declared circulatory death and initiation of warm ischemia when asystole or systolic blood pressure below 30 mmHg was observed (14, 16–18). Niederberger et al. suggested that a clear definition of circulatory arrest and the timing of warm ischemia was significant because damage for the grafts before procurement might vary dramatically according to the different definitions of the warm ischemia start time (3).

In the present study, we set up a clinically relevant and reproducible rat model of cardiac DCD combined with normothermic ESHP. The highlights of our model could be elaborated as follows. Firstly, we adopted the current cardiac DCD protocol (14, 16), including tracheal clamping to induce asphyxiation, as well as the definition and duration of WIT to increase the reproducibility of the cardiac DCD model.

Secondly, to simulate the real-world practice, we established the normothermic ESHP system for rats with a peristaltic pump, membrane oxygenator, blood reservoir, infusion syringe pump, and water-bath box. The water-bath box containing water, heater, stirrer, and temperature-controlling switch was built to maintain the temperature of the isolated heart at 35–37°C. Besides, we set up the circuit with sterile Tygon tubing to avoid the impact of bacteria. Although silicon tube was the first choice for the peristaltic pump, we found in preliminary studies that the Tygon tubing was comparable to silicon tube in our experimental settings.

Thirdly, in our ESHP system, we chose a modified blood-based perfusate with a total volume of 18 ml, including 10 ml blood and 8 ml modified Krebs-Henseleit solution. The high oxygen-carrying capacity of hemoglobin can guarantee an adequate supply of oxygen to the myocardium. The coronary flow rate can also be restricted at a physiological level (2–3 ml/min/g heart weight), avoiding the negative impact of hyperperfusion caused by crystalloid solution on the endothelium of coronary arteries (25). Taking into account calcium binding to the circulating protein in the blood, we increased the content of calcium in the Krebs-Henseleit solution to maintain a higher ionized calcium concentration (1.1 mM at the beginning of perfusion) in the perfusate. We also supplemented the perfusate with mannitol to relieve myocardial edema and methylprednisolone sodium succinate to decrease the inflammatory response, considering the diluted allogenic blood without leukocyte depletion (34). Fourthly, to support the metabolism and function of DCD hearts during ESHP, we administered insulin and epinephrine into the perfusate and maintained the PH and glucose level of perfusate according to blood gas analysis.

Fifthly, our ESHP system allowed us to perform a cardiac functional evaluation through a latex balloon to assess the transplantability of DCD hearts. Finally, our well-established rat model of cardiac DCD combined with ESHP is efficient, reproducible, and economical for the study of technical and practical issues in ESHP as the total cost of our ESHP system is within 5,000 dollars. Furthermore, the learning curve of our model was rather smooth as the researchers could become skilled with this model by practicing as little as 5 experiments.

There are several limitations in our present study. Firstly, the perfusate level of lactate increased over time during ESHP, especially in the non-DCD group. Although the perfusion flow rate was chosen within the physiological coronary flow rate, relative hypoperfusion for the isolated hearts may exist under the ESHP conditions regarding the perfusion pressure and retrograde perfusion, which was different from the physiological conditions. The relative hypoperfusion may account for the increase of lactate in both groups. Besides, the better cardiac function and more active metabolism in hearts without warm ischemia may lead to the higher lactate level during ESHP under the relative hypoperfusion. Future studies are necessary to determine the optimal coronary flow rate for the isolated hearts under such normothermic ESHP conditions. Secondly, we set up our ESHP system with a peristaltic pump, which inevitably leads to ongoing hemolysis. To minimize the influence of hemolysis, we expanded the volume of perfusate by using allogenic blood without leukocyte depletion. In our preliminary studies, we observed obvious hemolysis and potassium concentration above 9 mmol/L after 2 h of ESHP, accompanied by the compromised contractile function of hearts. Therefore, we suggested the hemolysis was the main factor limiting the ESHP duration and compromising the cardiac function during prolonged ESHP. 90 min might be the optimal duration of ESHP in our model. Centrifugal pump and expanding the volume of blood-based perfusate by using allogenic blood from two or more rats are suggested to attenuate the hemolysis in future studies. Thirdly, the closed-loop design of our ESHP model and the absence of livers and kidneys might contribute to an accumulation of metabolic waste and electrolyte imbalance in the perfusate, which was related to the functional performance of perfused hearts. Further studies are needed to investigate the approaches and effects of clearing the metabolic waste and adjusting the electrolyte imbalance. Fourthly, neurologic injury has not been considered in our cardiac DCD protocol. The neurologic injury occurs in the majority of DCD donors in clinical practice and can result in hemodynamic instabilities and further damage the donor heart (29). Finally, in the present study, rats were exposed to isoflurane, which might impact the generality of our results since isoflurane was cardioprotective and not generally used in clinical practice. Injectable anesthetics might be more advisable for the rat model of cardiac DCD (3, 35).

## Conclusions

In conclusion, we have established a clinically relevant, reproducible, and economical rat model for cardiac DCD combined with normothermic ESHP, where the DCD hearts can maintain a stable state during 90-min ESHP. This novel model will help to solve some key issues regarding ESHP at a reasonable cost in the upcoming studies before the efficient clinical translation for ESHP, thereby facilitating the development of novel therapeutic strategies to increase the quality of DCD hearts and expand the donor pool in the hearttransplantation.

## Ethics Statement

The animal study was reviewed and approved by the Ethical Committee of the Laboratory Animal Research Center of Southern Medical University Nanfang Hospital.

## Author Contributions

PZ raised the idea of this study. KL and SZ designed the prototype of this normothermic *ex situ* heart perfusion system. PZ, KL, and SZ designed the experiments and revised the manuscript. SZ provided the research funding for the project. JL and YX built up and improved the normothermic *ex situ* heart perfusion system. JL, YX, and CX performed the animal experiments. XL and CX performed molecular biology experiments. JL, XL, CX, and YX participated in the analysis of data and the writing of the manuscript. DP, HC, SL, and QP helped to conduct the experiments and collect the data. The authors read and approved the final manuscript. All authors contributed to the article and approved the submitted version.

## Conflict of Interest

The authors declare that the research was conducted in the absence of any commercial or financial relationships that could be construed as a potential conflict of interest.

## Publisher's Note

All claims expressed in this article are solely those of the authors and do not necessarily represent those of their affiliated organizations, or those of the publisher, the editors and the reviewers. Any product that may be evaluated in this article, or claim that may be made by its manufacturer, is not guaranteed or endorsed by the publisher.

## Abbreviations

ATP: Adenosine triphosphate
DAPI, 4′: 6-diamidino-2-phenylindole
DCD: Donation from circulatory death
DP: Developed pressure
dP/dt_max_: Maximum rate of rise of left ventricular pressure
dP/dt_min_: Maximum rate of pressure decline
ESHP: *Ex situ* heart perfusion
GSH: Glutathione
HNE: 4-Hydroxynonenal
HR: Heart rate
HTx: Heart transplantation
HCT: Hematocrit
IL-6: Interleukin-6
IOD: Integrated optical density
IRI: Ischemia/reperfusion injury
KHS: Krebs-Henseleit solution
MDA: Malonaldehyde
SBP: Systolic blood pressure
SOD: Superoxide dismutase
TNF-α: Tumor necrosis factor-α
TTC, 2, 3: 5-triphenyl-tetrazolium chloride
TUNEL: Terminal deoxynucleotidyl transferase dUTP nick end labeling
WIT: Warm ischemia time.
