# Supplementary material for: A Novel Rat Model of Cardiac Donation After Circulatory Death Combined With Normothermic ex situ Heart Perfusion
Source: Front Cardiovasc Med. 2021 Jul 23;8:639701. doi: 10.3389/fcvm.2021.639701 (PMC8342755; doi:10.3389/fcvm.2021.639701)
Supplement: Supplementary file 1 [file Image_1.pdf]

Supplemental materials

Supplementary Figure 1. The negative and positive control for the TUNEL staining of myocardial tissue.

(A) Negative control (magnification of 20; scale length: 50 μm); (B) Positive control (magnification of 20; scale length: 50 μm). Myocardial tissue treated with DNase was used as positive control and those without treatment of terminal deoxynucleotidyl transferase enzyme as negative control. DAPI: 4′,6-diamino-2-phenylindole (DAPI, blue); TUNEL: terminal deoxynucleotidyl transferase-mediated dUTP nick end-labeling.

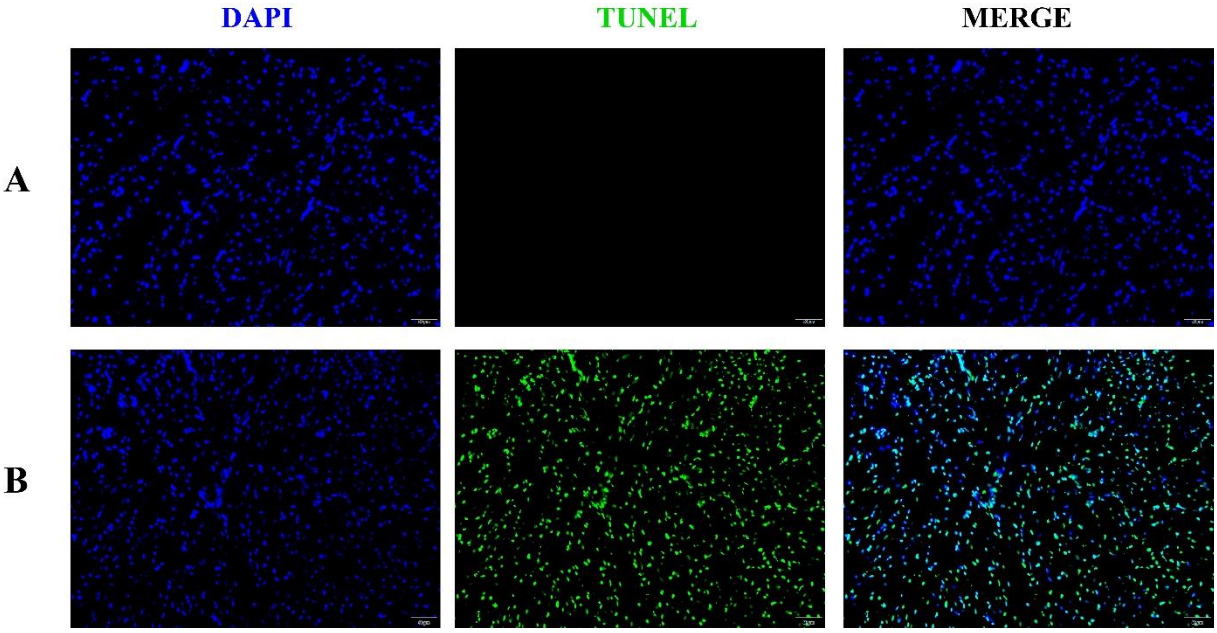
DAPI
TUNEL
MERGE
A
B
